# A Case of Cardiac Tamponade in a Patient with Metastatic Renal Cell Carcinoma on Pazopanib Treatment

**DOI:** 10.1155/2020/9268923

**Published:** 2020-04-09

**Authors:** Vinu Sarathy, Sriniivas Belagutty Jayappa, Thianesh Waran, Radheshyam Naik

**Affiliations:** Department of Medical Oncology, HealthCare Global Enterprises Ltd., Bangalore, India

## Abstract

Asymptomatic minimal pericardial effusion may be frequently found in patients with hypothyroidism. Cardiac tamponade secondary to hypothyroidism is rarely referenced in medical literature. Hypothyroidism as an adverse effect of pazopanib (tyrosine kinase inhibitor) treatment leading to cardiac tamponade is an even rarer occurrence. Here, we report an unusual case of a 71-year-old male, with a case of renal cell carcinoma on pazopanib treatment presenting with shortness of breath who was found to have hypothyroidism with a large pericardial effusion leading to cardiac tamponade. The patient did not have any prior reports of thyroid-stimulating hormone (TSH) or thyroid hormone levels at presentation. No such case of cardiac tamponade due to hypothyroidism as an adverse effect of pazopanib tablet treatment has been reported to our knowledge.

## 1. Introduction

Pericardial effusion and cardiac tamponade are caused due to a variety of etiological factors which include acute pericarditis, tumor, uremia hypothyroidism, trauma, cardiac surgery, or other inflammatory/noninflammatory conditions.

Recent studies have shown that pericardial effusions are extremely rare in hypothyroidism with an incidence of 3 to 6 percent [1].

Patients who are on pazopanib have been reported to develop hypothyroidism at an incidence of less than 10% [2].

A small pericardial effusion can cause significant cardiac tamponade when it accumulates rapidly, and hence, it is important to suspect cardiac tamponade in patients with sudden hemodynamic compromise [3].

Here, we report an uncommon case of hypothyroidism with cardiac tamponade.

## 2. Case Report

A 71-year-old male patient who was a known case of metastatic renal cell carcinoma on treatment with the tablet pazopanib 800 mg OD since 5 years presented to us for the first time in ER on 19.06.2018 with complaints of shortness of breath and easy fatigability.

On examination, the patient was breathless and had a sallow complexion with dry skin and peripheral edema with prominent visible neck veins.

The patient's blood pressure was 100/70 mmHg with tachycardia and oxygen saturation of 92% at room air. The patient had demonstrable pulsus paradoxus with an inspiratory drop in a blood pressure of more than 16 mmHg.

**Figure 1:**
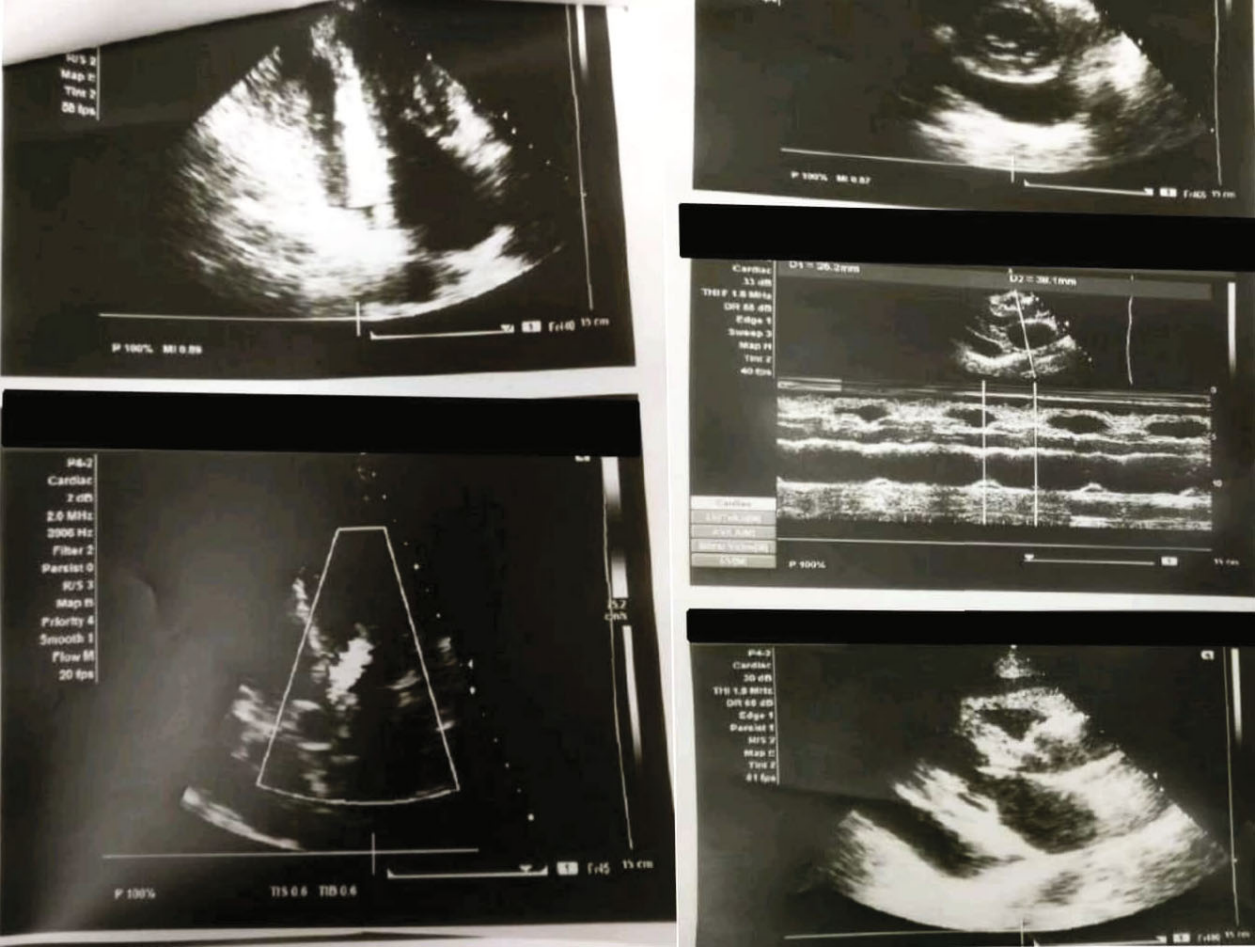
2D ECHO showing a large pericardial effusion with right atrial diastolic collapse suggestive of cardiac tamponade.

Bedsides, a 2D echocardiogram showed a large pericardial effusion with right atrial right ventricular diastolic collapse suggestive of cardiac tamponade as shown in Figure 1.

A primary working diagnosis of suspected disease progression with pericardial/cardiac metastases was made.

The patient was immediately shifted to the Intensive Care Unit and underwent pericardiocentesis. About 350 ml of yellowish golden colour fluid was aspirated which was sent for cytological and biochemical investigation.

Immediately post procedure, the patient showed significant improvement in cardiopulmonary status. His tachypnea and tachycardia subsided, and the patient maintained adequate oxygen saturation on room air. Post pericardiocentesis, the 2D echocardiogram revealed minimal pericardial fluid with no evidence of cardiac tamponade.

Pericardial fluid analysis showed degenerate and mesothelial cells and no evidence of any malignancy or acid fast bacilli.

**Figure 2:**
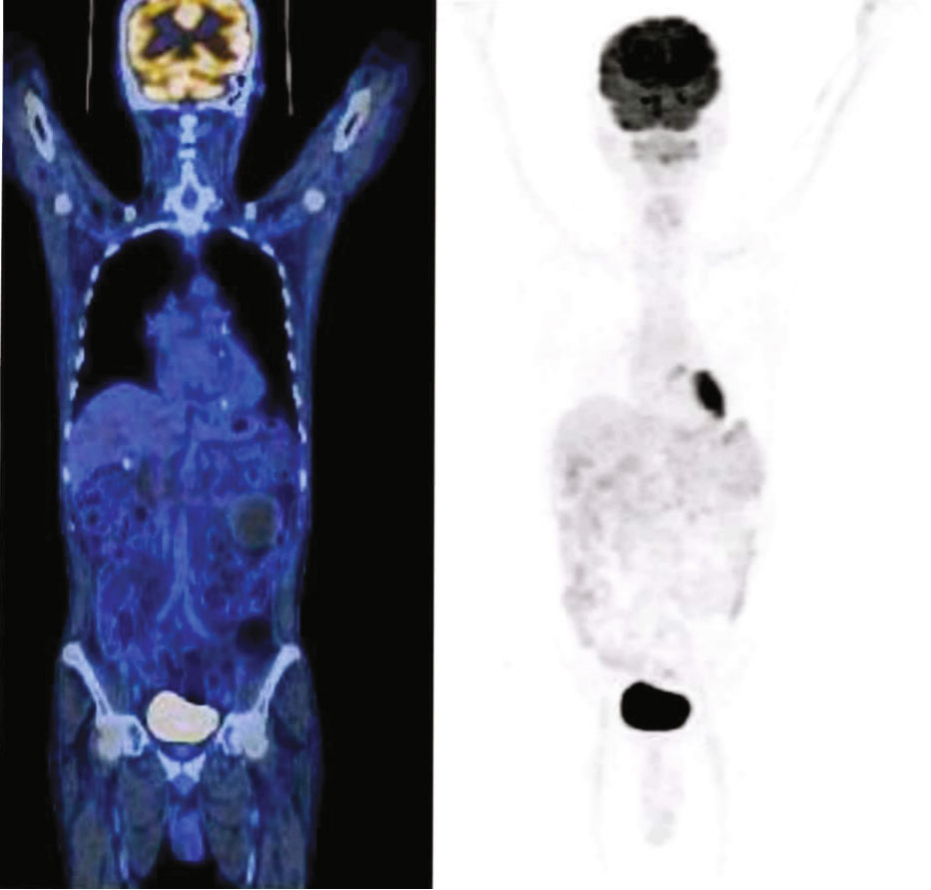
18-FDG whole body PET CT showing stable disease and metabolic activity.

However, to rule out disease progression, the patient had an 18-fluorodeoxyglucose (FDG) PET-CT scan (Figure 2) which showed regression of the necrotic mass of the left kidney, stable proximal left renal tumor thrombosis, stable lesion adjacent to tumor thrombosis in the anterior calyx, stable metastatic right lung nodule, and interval development of bilateral pleural effusions and mild to moderate pericardial effusion with pericardial catheter drainage tube in situ.

Considering his history and findings, we did a thyroid function test which was suggestive of severe hypothyroidism with high TSH levels (TSH > 100 mIU/L) and low T3 T4 levels.

The patient did not have any prior reports of TSH or thyroid hormone levels. Antithyroid peroxidase antibody testing was done which was negative.

The patient was treated with the tablet levothyroxine 50 micrograms daily which was later gradually increased to 125 mcg daily. The patient's condition improved significantly and was discharged in a stable condition 1 week post removal of the pericardial drainage tube.

Follow-up echocardiogram after a period of 2 months showed near total resolution of pericardial effusion. Within a few weeks, all his signs and symptoms completely resolved and the patient is currently continuing treatment on pazopanib tablet as part of his renal cell carcinoma treatment.

The patient is on regular follow-up, and his latest 2D Echo on 10.10.2018 showed minimal pericardial effusion with good systolic left ventricular function. The patient is currently hale and hearty on thyroid replacement and pazopanib tablet treatment with a normalized TSH value of 0.97 mIU/L.

## 3. Discussion

Cardiac tamponade as a complication of hypothyroidism is very rare with few cases described in world literature [4].

Hypothyroidism may commonly cause asymptomatic pericardial effusions, but rarely leads to symptomatic tamponade. The mechanism of this myxomatous pericardial effusion is postulated to be due to the increased permeability of capillaries causing leakage of fluid rich in protein into the interstitial space and impaired lymphatic drainage leading to salt and water retention [5].

Although the definitive mechanism of tyrosine kinase inhibitor- (TKI-) induced hypothyroidism is debatable, several theories have been proposed.

Vascular endothelial growth factor (VEGF) is a signal protein allowing formation of new blood vessels as well as formation of collateral circulation. TKIs like pazopanib reduce blood supply to the tumor leading to tumor regression; however, capillary regression is also noted in normal organs like the thyroid considering that the thyroid is an organ with the highest blood flow per unit weight [6].

Other mechanisms proposed include alterations in thyroxine/triiodothyronine metabolism [7], induction of type 3 deiodinase activity [8], blockage of iodine uptake [9], and autoimmunity where antithyroid peroxidase antibodies are detectable [10].

However, the latter seems highly unlikely in our patient as antithyroid peroxidase antibodies were not detected.

Another interesting extrapolation which our patient may have raised is whether adverse effects of treatment (severe hypothyroidism in our patient) translate into increased clinical benefit.

Pazopanib treatment in advanced renal cell carcinoma has been known to provide an average of 9 months of progression-free survival and 23 months of overall survival. Our patient has shown a progression-free survival of 5 years till date which is remarkable considering the much lower average.

The above has been demonstrated in previous studies showing hypothyroidism and other adverse effects of TKIs improved tumor response rates and survival time of kidney cancer patients treated with targeted therapy [11].

During pazopanib therapy, monitoring the thyroid function must be necessary.

Our case also highlights further the importance of stringent follow-up and monitoring to pick up adverse effects at the earliest time and prevent potentially life-threatening events.

## 4. Conclusion

Atypical symptoms arising in cancer patients on treatment should provoke other more easily treatable differential diagnoses like treatment-related adverse events rather than just disease progression.

Treatment-related adverse events may translate into a better response and survival rate for the patient.
